# Ebola haemorrhagic fever outbreak in Masindi District, Uganda: outbreak description and lessons learned

**DOI:** 10.1186/1471-2334-11-357

**Published:** 2011-12-28

**Authors:** Matthias Borchert, Imaam Mutyaba, Maria D Van Kerkhove, Julius Lutwama, Henry Luwaga, Geoffrey Bisoborwa, John Turyagaruka, Patricia Pirard, Nestor Ndayimirije, Paul Roddy, Patrick Van Der Stuyft

**Affiliations:** 1 Unit of Epidemiology and Disease Control, Institute of Tropical Medicine, Antwerp, Belgium; 2 Infectious Disease Epidemiology Unit, London School of Hygiene & Tropical Medicine, Keppel Street, Bloomsbury WC1E 7HT, London, UK; 3 District Directorate of Health Services, Masindi, Uganda; 4 MRC Centre for Outbreak Analysis and Modelling, Imperial College, London, UK; 5 Uganda Virus Research Institute, Entebbe, Uganda; 6 Médecin Sans Frontières, Brussels, Belgium; 7 World Health Organisation African Region, Kampala, Uganda; 8 Médecin Sans Frontières, Barcelona, Spain; 9 Institute of Tropical Medicine and International Health, Charité - Universitätsmedizin Berlin, Spandauer Damm 130, D-14050, Berlin, Germany; 10 National Tuberculosis and Leprosy Programme, Ministry of Health, 2 Lourdel Road, Wandegeya, P.O. Box 16069, Kampala, Uganda; 11 Masindi District Health Office, Box 67, Masindi, Uganda; 12 World Health Organization Liberia, Monrovia, Liberia

## Abstract

**Background:**

Ebola haemorrhagic fever (EHF) is infamous for its high case-fatality proportion (CFP) and the ease with which it spreads among contacts of the diseased. We describe the course of the EHF outbreak in Masindi, Uganda, in the year 2000, and report on response activities.

**Methods:**

We analysed surveillance records, hospital statistics, and our own observations during response activities. We used Fisher's exact tests for differences in proportions, t-tests for differences in means, and logistic regression for multivariable analysis.

**Results:**

The response to the outbreak consisted of surveillance, case management, logistics and public mobilisation. Twenty-six EHF cases (24 laboratory confirmed, two probable) occurred between October 21st and December 22nd, 2000. CFP was 69% (18/26). Nosocomial transmission to the index case occurred in Lacor hospital in Gulu, outside the Ebola ward. After returning home to Masindi district the index case became the origin of a transmission chain within her own extended family (18 further cases), from index family members to health care workers (HCWs, 6 cases), and from HCWs to their household contacts (1 case). Five out of six occupational cases of EHF in HCWs occurred after the introduction of barrier nursing, probably due to breaches of barrier nursing principles. CFP was initially very high (76%) but decreased (20%) due to better case management after reinforcing the response team. The mobilisation of the community for the response efforts was challenging at the beginning, when fear, panic and mistrust had to be countered by the response team.

**Conclusions:**

Large scale transmission in the community beyond the index family was prevented by early case identification and isolation as well as quarantine imposed by the community. The high number of occupational EHF after implementing barrier nursing points at the need to strengthen training and supervision of local HCWs. The difference in CFP before and after reinforcing the response team together with observations on the ward suggest a critical role for intensive supportive treatment. Collecting high quality clinical data is a priority for future outbreaks in order to identify the best possible FHF treatment regime under field conditions.

## Background

Since its discovery 1976 in Nzara and Maridi, Sudan [1], the Sudan ebolavirus (SEBOV) has caused three further epidemics in humans: Nzara, Sudan, 1979 [2], Gulu, Mbarara and Masindi, Uganda 2000 [3-5], and Yambio, Sudan, 2004 [6]. The reservoir of SEBOV and the mode of primary transmission to man are unknown. Secondary spread occurs through direct contact with infected patients, their body fluids or remains.

The 2000 Ebola haemorrhagic fever (EHF) outbreak in Uganda was first recognised on 8 October by the health authorities of Gulu district [4]. A national and international response was swiftly organised. On two occasions, infected individuals travelled from Gulu to other districts in Uganda, becoming the origin of secondary outbreaks: in Mbarara (5 cases), 620 km south of Gulu [7,8], and in Masindi (26 cases), 170 km south of Gulu. In January 2001, the outbreak's final case was detected in Gulu [9]. With a total of 425 cases, the EHF outbreak in Uganda in 2000-2001 is the largest described to date [3-5].

This paper focuses on the secondary EHF outbreak in Masindi district. We describe the course of the outbreak, report on response activities, and attempt to appreciate strengths and weaknesses of the outbreak response. Albeit limited in the number of infected individuals, the outbreak control measures were met by a number of significant challenges, which merit scrutiny for improving future efforts.

## Setting and methods

### Setting

**Figure 1:**
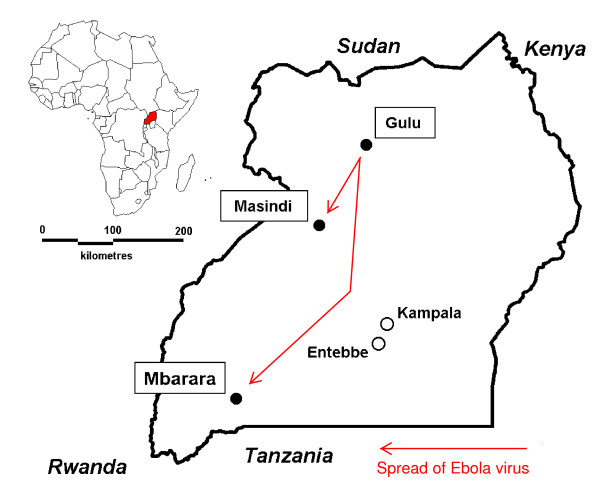
**Gulu, Masindi and Mbarara in Uganda**. The map shows the location of the epicentrum (Gulu) of the EHF outbreak in Uganda, 2000, and of the locations were satellite outbreaks occurred (Masindi, Mbarara).

Masindi district is a rural district with a population of 400,000 at the time of the outbreak; agriculture and sugar industry are its main economic activities. Masindi town (Figure 1), the district's administrative centre, had at that time a population of 25,000. The main road from Uganda's capital Kampala to the country's northern districts traverses the district but not Masindi town. Masindi district is characterised by its ethnic diversity: the population consists of 56 ethnic groups, of which the Banyoro and the Bagungu together form a 60% majority. Immigrants and refugees from neighbouring countries have settled in this region for decades. As elsewhere in Uganda, the five-tier local government structure consists of elected Local Councils, headed by a chairman.

The governmental health system of Masindi district is headed by the District Director of Health Services (DDHS) in Masindi town. The district has 37 health centres and two hospitals: Masindi hospital (78 beds) and Kiryandongo hospital (104 beds). Kiryandongo hospital has more beds but is less well equipped, staffed and frequented.

### Data sources and methods

We analysed surveillance records, hospital statistics and our own observations during response activities. While some clinical data could be extracted from surveillance records, clinical dossiers of individual patients were not available for analysis. We used two-sided Fisher's exact tests for differences in proportions, two-sided t-tests for differences in means, and logistic regression for multivariable analysis.

The National Task Force for the Gulu Ebola Outbreak authorised data collection and publication. No additional ethics approval was necessary, since we used data collected routinely in an emergency situation. Patients, relatives and health care workers were informed that a report would be published, and that their privacy would be respected. Permission to use the data for publication was granted by the Ministry of Health, whose local representatives at the time are among the co-authors of this paper.

## Results

### Onset of the outbreak

On October 27, 2000, a representative of the local government notified the sub-district health office of a suspected EHF death. The individual, a woman in her sixties, had been treated in St. Mary's Hospital Lacor in Gulu for an unrelated chronic condition but had left the hospital and returned home to Kaduku II hamlet, Masindi district, after one of the nurses, who had treated her in Lacor Hospital, had died from EHF. The woman reportedly fell ill on October 21st and died at home on October 25. Her extended family, including relatives from Kenya, and many neighbours attended her funeral.

On November 7, the sub-district office received information that two further members of the same family had died. The next day, the medical superintendent of Kiryandongo hospital visited the family and found that her three-week-old grandson and a thirty-year-old daughter had died some days before, and that her 70-year-old husband was ill with symptoms compatible with EHF. The husband was transferred to Kiryandongo hospital, placed in isolation, and had a blood sample taken. EHF was confirmed on November 12. This is when the District Task Force was established, and national and international resources were mobilised to respond to the outbreak.

### Outbreak response

#### Co-ordination

The outbreak response was co-ordinated locally by the District Response Task Force, presided by the Chairman of the Local Council at district level. The Task Force met twice weekly and united all concerned sectors of the society with representatives from international organisations and foreign institutions involved in the response. The Task Force's technical committee, led by the DDHS, consisted of local, national and international experts on surveillance, case management, logistics and public mobilisation. It met daily, reporting on activities and their outcomes, establishing up-to-date case lists, providing cumulative figures of cases and deaths to the Ministry of Health (MoH) and planning for the following day. Subcommittees for surveillance, case management, logistics and public mobilisation planned and implemented the daily activities. Although time-consuming, these daily meetings played a crucial role in ensuring that all response aspects were covered while avoiding the duplication of efforts, and in fostering mutual trust and confidence among intervening partners.

#### Case definitions

The four case definitions developed and employed in Gulu were also applied in Masindi. Each definition served a specific group of individuals to take a specific decision (Table 1). From 'alert' via 'suspect' and 'probable' to 'confirmed', specificity increased at the cost of simplicity. All case definitions were meant to be highly sensitive because of the detrimental consequences of not identifying an EHF patient.

**Table 1:** Ebola haemorrhagic fever case definitions, Masindi/Uganda 2000

Term	Used by	To decide on	Criteria
Alert case	Community members, health care workers not directly involved in EHF response	Alert mobile surveillance team?	A1 Any person with sudden onset of fever A2 Any person with haemorrhage A3 Any sudden death

Suspect case	Mobile surveillance team	Transport to hospital?	S1 Any person who [had slept in the same house as a case, or had touched the body of a case (dead or alive), or had touched linens or body fluids of a case] AND has fever S2 Any person who has at least three of the following symptoms: [headache, nausea/vomiting, loss of appetite, diarrhoea, intense fatigue, abdominal pain, muscle or joint pain, difficulty with swallowing, difficulty with breathing, hiccup] AND has fever S3 Any person with any of the following symptoms: bleeding gums, bleeding into the eyes, bleeding into the skin, black or bloody stool, bloody vomit, nose bleed

Probable case	Clinicians of isolation unit	Isolate patient and take blood sample for EHF testing?	*As for suspect case, plus not explicitly defined clinical criteria for differential diagnoses*

Confirmed case	Clinicians of isolation unit	Transfer patient to confirmed case section within Ebola ward?	C1 A probable case with a positive Ebola antigen capture ELISA or PCR C2 A probable case with prior contact to another confirmed case and positive for anti-Ebola IgG ELISA

A 'suspect case' was an individual with a combination of certain symptoms and exposures (Table 1, case definitions S1-3). A suspect case for whom an experienced clinician has ruled out alternative diagnoses was classified as 'probable case'. A 'laboratory-confirmed case' was a probable case with a positive antigen-capture ELISA or PCR result (Table 1, C1) [10], or a probable case with prior contact to another confirmed case and a positive IgG ELISA result (Table 1, C2) [11]. IgM ELISA was attempted but results were not available during the outbreak in Masindi. Laboratory tests were performed in the CDC field laboratory in Gulu. Samples reached that laboratory usually within 24 h, and results were received mostly within 48 h.

#### Surveillance

Members of the public reported alert cases to the nearest health facility or the local government representative, who forwarded the alert to the District Health Office. Alerts were recorded in a "rumour registry", and responded to by members of the District's mobile surveillance team, who had been trained to investigate alerts, follow-up contacts, and recognise suspect cases of EHF. Any individual corresponding to the suspect case definition was transported by ambulance to Masindi hospital under barrier nursing precautions. All contacts (anyone having had physical contact with a suspect case or his remains, body fluids or soiled materials, or having lived in the same house as the case) were registered by the surveillance team and followed-up daily for 21 days, or until the suspect case was declared a non-case following negative clinical assessment or laboratory results.

In the vicinity of Masindi town, up to five teams of surveillance officers moved around in pairs on motorbikes, while farther away teams of four officers plus driver used a four-wheel drive vehicle. Teams were composed in such a way that at least one senior officer was present in each team. Their case identification decisions and activities were discussed with a local supervisor in daily surveillance sub-committee meetings. For communication with the District Health Office about alerts and transports to the hospital, very high frequency radio and cellular telephone were used.

#### Case management

At Masindi hospital, neither an isolation ward nor an unused ward were available. A general ward was therefore evacuated to be used exclusively for probable and confirmed EHF patients. When the number of EHF cases increased, a second general ward was incorporated in the extended Ebola ward.

**Figure 2:**
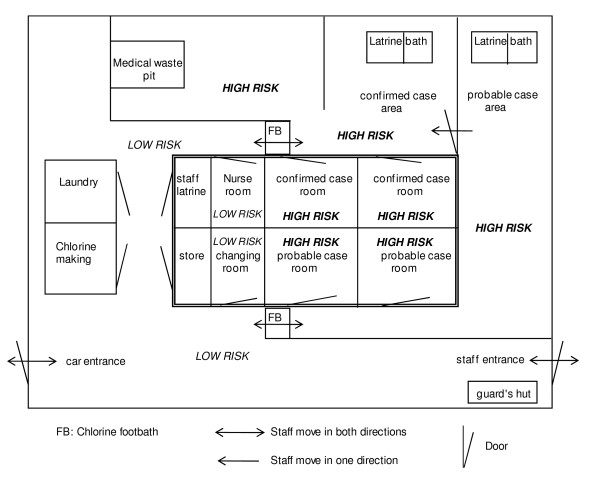
**Ebola Ward, Masindi General Hospital (before extension)**. The Ebola ward in Masindi corresponds to the typical set-up of FHF isolation wards consisting of three separate compartments: low risk for staff, storage; high risk for probable cases; high risk for confirmed cases; decontamination stations between compartments.

On arrival at Masindi hospital, a suspect case was brought to the screening area, established in a tent in front of the Ebola ward, where an experienced physician clinically assessed the individual. Based on interview and physical examination, the patient was either classified as 'probable case' and admitted to the probable case section of the Ebola ward (Figure 2), or as 'non-case' and admitted to the general ward for further diagnosis and treatment. Because of limited laboratory capacity only probable cases had a blood sample taken and sent to the temporary BSL 4 laboratory in Gulu for EHF diagnostics.

Due to space constraints of the ward, several patients were often hospitalised in the same room. Hospital staff were instructed to disinfect equipment and their gloves with 0.05% chlorine solution between contacting one patient and the next. Movements of the patients themselves, however, could not always be controlled. To further reduce the risk of transmission of SEBOV from a confirmed case to a probable case who would later turn out to be negative, probable and confirmed cases were kept in separate sections of the ward.

Hospital staff wore personal protective equipment (PPE) when caring for suspect, probable or confirmed cases; there was a strictly defined sequence of donning and removal of PPE according to WHO guidelines [12]. Staff moving from high to low risk areas stepped into a 0.5% chlorine foot bath, then disinfected and removed their PPE. Medical treatment was supportive and consisted of oral rehydration, analgesics and sedative drugs, and occasionally intravenous fluid replacement. Burials of deceased patients were undertaken using barrier nursing conditions. Traditional practices that involved direct contact with the corpse (washing the body etc.), were suspended throughout the outbreak.

#### Public mobilisation

Public mobilisation involved a variety of approaches. For addressing the communities, the support of the chairmen of local councils at sub-district and village level was sought and granted. Meetings with local council members and religious leaders were held prior to the public mobilisation teams directly addressing the community. Communication strategies for the community included speeches at local gatherings - sometimes specially convened, e.g. for traditional healers, sometimes making use of gatherings that served other purposes, e.g. religious services, video screenings, drama groups, radio spots, newspaper articles, posters and pamphlets. Red Cross volunteers undertook house-to-house mobilisation.

### Course of the epidemic

The first 2 weeks of the epidemic in Masindi were relatively calm, the situation apparently under control, and the first team of international experts prepared to return to Gulu where the epidemic was still very active. Towards the end of November the situation in Masindi deteriorated rapidly, and the international team was reinforced on December 7 by a delegation of international experts and experienced national HCWs from Gulu.

On 19 November 2000, 1 week after the outbreak had been declared, patients started to be admitted to the Ebola ward at Masindi hospital. All except the first three cases (n = 23) were treated in the Ebola ward of Masindi Hospital. The first death in the Ebola ward occurred on 24 November. Initially the work on Ebola ward suffered from staff shortage and low staff retention. The situation improved when the Masindi team was reinforced by HCWs from Gulu with significant experience working on Ebola response there.

**Figure 3:**
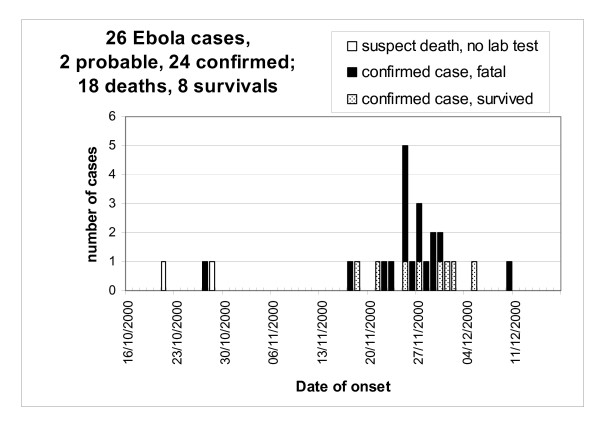
**EHF outbreak, Masindi district, Uganda, 2000**. The graph shows the number of laboratory confirmed Ebola cases over the course of the outbreak. Fatal and non-fatal cases are indicated.

The EHF epidemic in Masindi district lasted 2 months, from the index case's onset of disease on October 21st until the death of the last case on December 22nd (Figure 3). The official total number of cases was 27. During a follow-up visit, however, index family members unanimously stated that one unconfirmed case, a three-week-old infant, had never been in contact with any infected individual. We therefore consider this death to be coincidental, and removed the infant from the case list. The reviewed total of cases is therefore 26. All except the index case and one of the two 2nd generation cases are laboratory confirmed (n = 24). The district was declared Ebola free on 25 January 2001, 42 days (i.e., double the maximum incubation period) following the confirmation of EHF in the last case.

### Case characteristics

In total, almost 200 individuals were placed under surveillance as contacts of EHF cases. Fifty-two suspect cases (Table 1) were identified by surveillance teams. Eight were deceased; two of these were subsequently classified as probable cases by epidemiological criteria. The 44 living suspect cases were transported to the Masindi Ebola ward and assessed by experienced physicians. Twenty-nine of them were classified as probable cases, isolated on the Ebola ward and bled for EHF testing in Gulu, which returned 24 positive results. Fifteen living suspect cases were considered as non-cases by experienced clinicians and treated according to their alternative diagnosis; eight of these, although not fulfilling the criteria of probable cases, had a blood sample taken without being isolated, which constituted a breach of the official policy. All eight had negative laboratory results.

The age of confirmed and probable cases (n = 26) ranged from 2 to 70 years, with a median of 27.5 years. Two thirds of the cases were male. In confirmed cases, the most common general symptoms at admission were fever (88%), loss of appetite (88%), intense fatigue (75%) and headache (71%); of hemorrhagic symptoms, only bleeding gums was present at admission (4.2%). The mean stay on the Ebola ward was 6.1 days for fatal cases (ranging from 2 to 13 days), 8.0 for survivors (2 to 11). Patients who turned out to be non-cases stayed on average 3.3 days on the Ebola ward.

### Transmission

**Figure 4:**
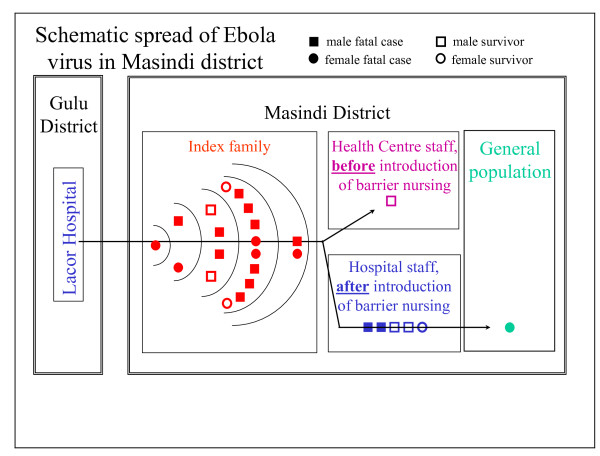
**Spread of Ebola virus in Masindi district, Uganda, 2000**. The graph shows the schematic spread of EBOV from the epicentre in Gulu to Masindi district, within the index family (five epidemiological gernerations of cases), to HCWs before and after the introduction of barrier nursing, and into the general population. Sex of the case and outcome of the disease are indicated.

The index case was likely infected from a HCW in Lacor hospital in Gulu. After returning home to Masindi district the index case became the origin of a transmission chain within her own extended family (18 further cases), from index family members to Masindi HCWs (6 cases), and from Masindi HCWs to their household contacts (1 case, Figure 4).

#### Transmission within the index family

**Figure 5:**
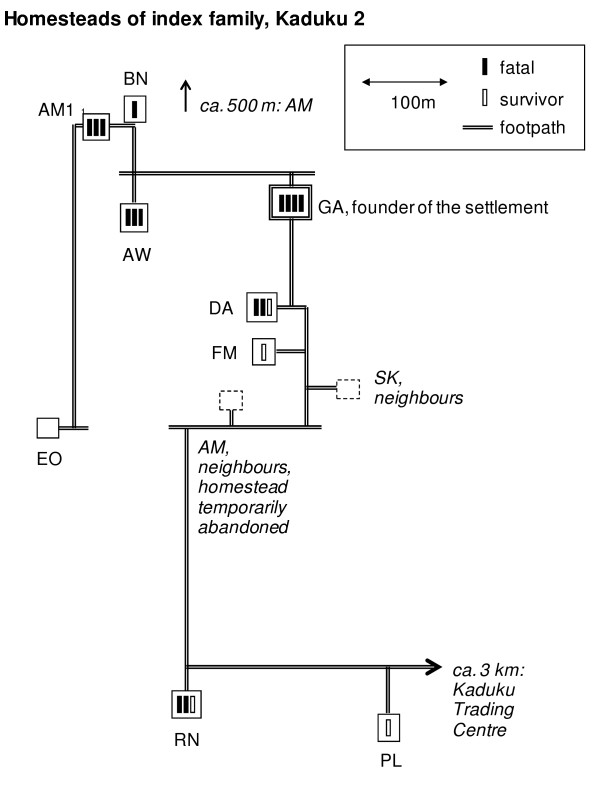
**Homesteads of index family, Kaduku II hamlet, Masindi district, Uganda, 2000**. The schematic map shows the locations of the index family's homesteads, with footpaths connecting them, and approximate distances between them. Fatal and survived cases are indicated. No cases occurred in homesteads not belonging to the index family.

**Figure 6:**
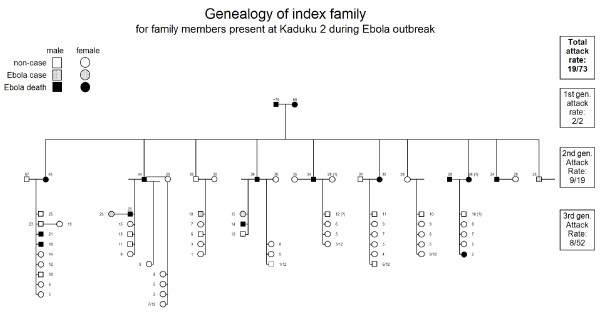
**EHF cases in family tree of index family, Masindi district, Uganda, 2000**. The graph shows the three genealogical generations of the index family, indicating sex, EBOV infection and EHF outcome. Attack rates per generation and in total are presented.

The 73 members of the extended index family, who had immigrated from Kenya decades before the outbreak, lived in Kaduku II, a hamlet scattered over an area of about 2 ha, intertwined with two unrelated households (Figure 5), close to Kaduku village. While these neighbours remained unharmed, several members of the extended family became infected, corresponding probably to 5 epidemiological generations of cases (Figure 4). In Figure 6, EHF cases and victims are plotted in the family tree. Both members of the founding generation died. The 2nd genealogical generation lost almost half of its members (8/19), leaving 4 full and 26 half orphans in school age or younger behind (< 15 years). The overall attack rate in the index family was 26% (19/73), but in the economically most active age group of 15 to 49 years, the attack rate was 53% (16/30). CFP in the index family was high (79%; 15/19). Because of multiple simultaneous contacts, transmission chains within the index family could not comprehensively established, but contact histories suggested the existence of a super-spreader by multiple contacts: of three male adult third generation cases, one could be associated with transmissions to ten 4th generation cases, while the others were associated with one or two or transmissions only.

#### Transmission within the community

During the first days of the EHF epidemic, before it was recognised and declared as such, the index family had normal everyday contacts with their neighbours. Furthermore, the burials of two individuals, carried out in the traditional manner before declaration of the epidemic, attracted a large number of neighbours, plus further family members from near and far, including a delegation from Kenya. Among neighbours and family members who immediately returned home after these burials, no suspect cases were detected by the surveillance teams in Masindi district and in Kenya during the 3-weeks follow-up.

After the EHF outbreak was declared in Masindi, the index family's autochthonous neighbours imposed a quarantine on the family, whose members were no longer allowed to leave the compound (Figure 5) except to fetch water. The health authorities accepted this quarantine as a contribution to contain the outbreak, as it reduced the number of contacts and concentrated them geographically; daily follow-up was thus logistically less demanding. During quarantine, the surveillance team observed only few movements on their daily visits between the index family's settlement and the neighbouring communities. No EHF cases were recognised in the communities surrounding the index family.

The only community EHF case outside the index family occurred in a housemaid working and giving nursing care in a HCW's household. This HCW had acquired EHF on the Ebola ward, had refused hospitalisation for a couple of days, and survived; the housemaid later died of EHF in the Ebola ward.

#### Occupational transmission to health care workers

Before the Masindi EHF outbreak was recognised, several index family members attended the local health centre in Kaduku village, where one of the nurses became infected (Figure 4). This was the only case of occupational EHF before barrier nursing procedures were introduced. Five more HCWs, however, all members of the EHF case management team, became infected after the declaration of the outbreak and the introduction of barrier nursing. The likely cause for these occupational EHF cases were violations of barrier nursing principles. According to fellow HCWs, such violations included: cleaning the ambulance without full protective gear after transporting suspect cases and smoking while doing so, washing soiled linen of patients without full protective gear on the Ebola ward, or answering the mobile phone while working in the contaminated section of the Ebola ward.

#### Nosocomial transmission

Beyond the above mentioned likely nosocomial transmission from a HCW in Gulu to the index case, nosocomial transmission likely also occurred in the Masindi Ebola ward, where a 2 years old child became infected after close contact with its hospitalised and infected mother; based on dates of onset, transmission in the household prior to hospitalisation cannot be ruled out but appears less likely.

### Performance of surveillance activities

Since no cases were observed outside community contacts under surveillance or HCWs, the alert case definition (Table 1) did not play a major role for the outbreak's containment. Table 2 summarises the presence of criteria for suspect cases in individuals classified by clinicians as probable or non-cases, as well as sensitivity and specificity of all criteria and different combinations, as well as the equivalent information for probable cases, classified by the CDC field laboratory in Gulu as confirmed or non-cases. In Masindi, where transmission occurred exclusively among identified contacts, it was desirable that the epidemiological/clinical case definition S1, which required contact plus fever only, was sufficient to identify all probable cases. Twenty-five out of 28 probable cases following the index case were identified through this case definition; case definitions S2 (at least three general symptoms plus fever) or S3 (any bleeding sign) did not add a single probable case. The combined case definition S (= S1 *or *S2 *or *S3) had thus a sensitivity of 89%, but a specificity of 7% only. Three individuals failed to fulfil the suspect case definition because they reported neither fever nor bleeding. The surveillance team, however, overruled the case definition on the basis of the individuals' prior contact to an EHF case and presentation of three to seven general symptoms. These individuals were judged by clinicians to be probable cases and were later identified as confirmed ones. One has to bear in mind, though, that most cases came from one extended family, so that HCWs using the case definition on its members had the benefit of an increased prior probability, which likely made the case definition more efficacious than in an outbreak with many transmission chains of unknown origin.

**Table 2:** Symptoms in suspect EHF cases by result of clinician's assessment, and in probable EHF cases by result of laboratory investigation

		Clinicians' assessment of suspect cases, n = 44*						Laboratory investigation of probable cases, n = 37					
		Probable, n = 29*			Non-cases, n = 15			Confirmed, n = 24			Non-cases, n = 13*		
	*Criteria*	+	-	Se.^#^	+	-	Sp.^§^	+	-	Se.^#^	+	-	Sp.^§^
	prior contact	28	1	97%	12	3	20%	24	0	100%	10	3	23%

	fever	25	3	89%	13	2	13%	21	3	88%	11	1	8%

*General signs:*	headache	20	8	71%	9	6	40%	17	7	71%	8	4	33%
	nausea/vomiting	11	17	39%	6	9	60%	10	14	42%	5	7	58%
	loss of appetite	22	6	79%	9	6	40%	21	3	88%	6	6	50%
	diarrhoea	11	17	39%	3	12	80%	11	13	46%	2	10	83%
	intense fatigue	19	9	68%	9	6	40%	18	6	75%	7	5	42%
	abdominal pain	17	11	61%	6	9	60%	15	9	63%	6	6	50%
	muscle or joint pain	13	15	46%	7	8	53%	13	11	54%	4	8	67%
	difficulty with swallowing	7	21	25%	1	14	93%	7	17	29%	1	11	92%
	difficulty with breathing	5	23	18%	0	15	100%	5	19	21%	0	12	100%
	hiccup	1	27	4%	1	14	93%	0	24	0%	2	10	83%

*Bleeding signs:*	bleeding gums	1	27	4%	0	15	100%	1	23	4%	0	12	100%
	bleeding into the eyes	0	28	0%	0	15	100%	0	24	0%	0	12	100%
	bleeding into the skin	0	28	0%	0	15	100%	0	24	0%	0	12	100%
	black or bloody stool	1	27	4%	1	14	93%	0	24	0%	2	10	83%
	bloody vomit	0	28	0%	3	12	80%	0	24	0%	1	11	92%
	nose bleed	1	28	3%	0	15	100%	0	24	0%	1	12	92%

*Case definitions:*													
S1	contact plus fever	25	3	89%	11	4	27%	21	3	88%	10	2	17%
S2	at least three general symptoms plus fever	21	7	75%	7	8	53%	19	5	79%	5	7	58%
S3	any bleeding sign	3	26	10%	4	11	73%	1	23	4%	4	9	69%
S2 or S3	any clinical case definition	21	7	75%	9	6	40%	19	5	79%	6	6	50%
S = S1 or S2 or S3:	any suspect case definition	25	3	89%	14	1	7%	21	3	88%	12	0	0%
P = S1 or S2 or S3 plus clin.ass.	probable = any suspect case definition plus clinical assessment							24	0	100%	5	8	62%

*Alternative case def.:^$^*													
S1a	contact plus (fever or 3+ general symptoms)							24	0	100%	10	2	17%
S2a	fever plus 2+ general symptoms							21	3	88%	9	3	25%
S2b	3+ general symptoms							22	2	92%	6	6	50%
S2c	2+ general symptoms							24	0	100%	10	2	17%

*: n < 29 due to missing clinical data for index case. ^#^: Sensitivity. ^§^: Specificity. ^$^: Alternative case definitions, not used during outbreak

Since all patients who were clinically assessed originated from contacts under surveillance or from HCWs, they should have been assessed on the date of onset or the following day. For calculating the delay between disease onset and clinical assessment we used as date of assessment either the date of admission or the date of first blood sample taken, whatever was the earlier date (Table 3). The mean delay for all 35 patients who were clinically assessed was 2.0 days, ranging from 0 to 8 days, the mean delay in 22 community cases (1.7 days) being shorter than in 13 HCW cases (2.6; *p *= 0.13, two-sided *t*-test). Only 37% (13/35) of patients were assessed on the day of onset or the next day as it would have been appropriate; 40% (14/35) were hospitalised on the 2nd day after onset, which may be considered acceptable, but for 23% (8/35) the delay was prolonged (≥ 3 days). Extreme delays (5 to 8 days) occurred in 3/13 HCW cases (23%), but did not occur in community cases (*p *= 0.044, two-sided Fisher's exact test). Causes for prolonged delays between onset and clinical assessment included, in community cases, missed daily visits by the surveillance teams and insufficient transport capacity, and, in both community and HCW cases, lack of cooperation by the individuals being followed-up, i.e. refusal to report symptoms or be taken to the hospital, as a result of fear or mistrust of the response team.

**Table 3:** Delays between onset and clinical assessment, and between admission to hospital and day of first blood sample, by professional background of patients

	Professional background of patients							
	**Community member**		**Health Care Worker**		**total**		***p*-value**	

Delay "onset of disease - clinical assessment" (days)	0-1	9	*41%*	4	*31%*	13	*37%*	
	2	9	*41%*	5	*38%*	14	*40%*	
	3-4	4	*18%*	1	*8%*	5	*14%*	
	5 or more	0	*0%*	3	*23%*	3	*9%*	
	Total	22	*100%*	13	*100%*	35	*100%*	*0.044
	mean	1.7		2.6		2.0		

Delay "admission to hospital - 1st blood sample taken" (days)	< 0 (before admission)	2	10%	5	63%	7	25%	**0.13
	0-1	14	70%	2	25%	16	57%	
	2	3	15%	0	0%	3	11%	
	3 or more	1	5%	1	13%	2	7%	
	total	20	100%	8	100%	28	100%	*0.009

*: Fisher exact test, two-sided, testing for an overall difference in proportions; **: unpaired *t*-test, two-sided, testing for a difference in means.

### Case fatality

The CFP in laboratory confirmed cases treated in Masindi for EHF was 64% (14/22), compared with 100% (4/4) in those who were not treated at Masindi Ebola ward (*p *= 0.28, Fisher's exact test, two-sided). Of the 22 confirmed EHF cases treated in Masindi for EHF, 15 were index family members, 6 were HCW who attracted EHF during work and thus were cases of occupational Ebola, and 1 HCW, not member of the response team, attracted EHF while caring for a diseased colleague at home.

**Figure 7:**
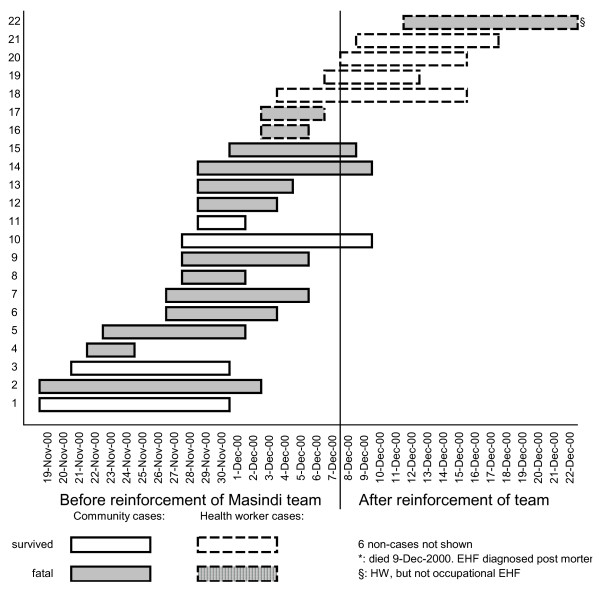
**Masindi Ebola ward: Patient flow (only confirmed EHF cases)**. The graph shows hospitalised survived and fatal EHF cases from the community and among HCWs, with date of admission and date of discharge/death, before and after the reinforcement of the outbreak response team.

Seventeen confirmed cases (15 community and two HCW cases) were treated entirely (n = 14) or mostly (n = 3) before the Masindi team was reinforced on December 7 (Figure 7). Five confirmed cases were treated entirely (n = 3) or mostly (n = 2) after reinforcement, all of them HCW cases (one non-occupational). The CFP in the first phase, prior to reinforcement, was 76% (13/17), and 20% (1/5) in the second phase (*p *= 0.039, Fisher's exact test, two-sided). Through logistic regression we found that the association between outbreak phase and survival persisted when controlling for age and sex (crude OR = 13, [95% CI 1.11-152]; adjusted OR = 16, [1.07-241]). The CFP was lower among patients with occupational EHF (33% [2/6]) than in non-occupational EHF (75% [12/16], *p *= 0.14), and lower in HCW cases than in community cases (43% [3/7] vs. 73% [11/15], *p *= 0.34).

### Burials

Safe burials were provided to all fatal cases from the Ebola ward. In Masindi, the morgue was initially not made available for the Ebola ward, as it was feared that this could create panic in dwellers of a settlement at 50 m distance, so that corpses remained on the ward until burial. This changed only after the arrival of the reinforcement from Gulu on December 7, when it was eventually accepted that corpses must be removed from the ward, and be taken to the morgue or be buried not later than on the day following the day of death.

Until December 4, safe burials were conducted in a reasonably timely fashion. On December 5 and 6, staff and community volunteers abandoned their work and four patients died within 2 days, so that a backlog of corpses built up on the Ebola ward. Before reinforcement from Gulu, 6/11 burials took place after an unacceptable delay, three of them on the 2nd day after death, the others on the 3rd day. After the arrival of reinforcement from Gulu, all three burials were carried out with acceptable delay.

To facilitate the mourning process and following good practice [13,14], Ebola victims were preferably buried close their families' homes. Adverse community reactions, however, forced the health authorities bury some victims on hospital grounds.

## Participant observations and discussion

Analysing surveillance records and hospital statistics allowed us to identify achievements and challenges in the EHF outbreak response activities in Masindi district.

### Achievements

With a single exception due to a HCW's refusal to be hospitalised, SEBOV did not invade the community at large (Figure 4). Instead, the outbreak at community level was limited to the extended family of the index case. The outbreak response efforts have, in all likelihood, significantly contributed to this containment. The acclaim for this should be shared between the outbreak response team for its vigorous efforts to trace cases and follow up contacts, and the community for imposing a quarantine on the index family. In our experience, international experts are usually reluctant to recommend quarantine measures, being concerned by human rights issues, fearing to antagonise the quarantined community, and arguing that quarantine is difficult to enforce. However, when the community itself imposes quarantine, its effectiveness may be more straightforward to ensure. Somewhat reluctantly the response team endorsed the quarantine, knowing that it would be difficult to convince the community to give it up, and hoping that it would not only decrease social mixing and exposure of susceptible individuals to SEBOV, but also reduce conflicts between the index family and their autochthonous neighbours. Surveillance officers convinced the community to allow the family to fetch water from the nearest well outside the family's compound, and made the quarantine more acceptable to the index family by providing food and supplies, which the family members were prevented from purchasing at Kaduku Trading Centre. Far from advocating community imposed quarantine as a standard outbreak response strategy, we acknowledge that in this specific outbreak it appeared to be effective in preventing the spread of SEBOV into the community at large, and in defusing tensions between the affected family and its neighbours.

A related achievement was the avoidance of transmission chains of unknown origin. While transmission could not always be prevented, when it occurred it was at least immediately clear where it had originated: all cases came from the population of contacts under surveillance. The absence of unknown contacts makes surveillance much easier, as the many cases of acute febrile illnesses without prior contact and without more specific symptoms are then unlikely to be EHF cases. This saves resources, and avoids feelings of being overpowered by the epidemic.

African traditions are likely to have prevented transmission to young children in the index family (Figure 6), the only paediatric EHF case probably originating on the Ebola ward. As described for other filoviral haemorrhagic fever (FHF) outbreaks, the tradition to keep young children away from ill family members seems to have protected the children from transmission of SEBOV [15-17].

The absence of nosocomial transmission to patients in Masindi, with the possible exception of a single transmission on the Ebola ward from mother to infant, was another achievement. This was again an effect of early case detection and isolation, which prevented EHF patients to seek care from unsuspecting HCWs in facilities other than the Ebola ward. Infection control on the Ebola ward was then sufficiently stringent to prevent SEBOV transmission to non-cases.

The mobilisation of the local government needs was successful. The local councils stood behind the disease control team unwaveringly, even during adverse community reactions. The community, sub-county and district level proved to be particularly important structures for the support of outbreak response activities: they liaised with the community including the index family, organised the cooperation with other sectors (education, environment etc.), and facilitated the collaboration between local authorities and the various international players.

Though the difference in CFP between patients who were hospitalised in Masindi and those who were not is statistically not significant, it suggests that hospital treatment of EHF might be of some use for the patient - that isolation is useful to halt an FHF epidemic is beyond doubt [12,18].

The presence of the field laboratory in Gulu has set a new standard for the control of major filoviral HF outbreaks. The availability of Ebola specific laboratory results within 24 to 48 h after blood sampling permits taking case management decisions based on laboratory confirmed diagnoses, while otherwise the role of the laboratory is limited to the concurrent or retrospective analysis of the epidemiological development. The result of a blood sample taken just after admission allows for the swift decision whether the patient needs to be isolated on the "confirmed" section of the Ebola ward, or whether he can be treated under normal conditions on a general ward. It is thus possible to reduce the risk of transmission to patients who have been isolated on clinical and epidemiological grounds but are de facto not EHF cases. A second test after clinical improvement allows for earlier discharge from the Ebola ward conditional on antigen clearance, thus assuring safety for contacts of convalescent patients, avoiding unnecessarily long stays in the isolation unit, decreasing stress levels for the convalescent, and reducing the work load for staff [19]. Clearly, to have a field laboratory on site is helpful to the outbreak control efforts.

In the aftermath of the outbreak, the hostile attitude towards the index family subsided and made way for expressions of solidarity. For instance, the local group of a service club organization donated school uniforms for the orphans and contributed to the acquisition of a maize mill to compensate for the loss of labour and to help the family generating some income.

### Challenges in the community

During times of crisis, the local tradition required that members of the index family stay even more closely together than they do anyway, taking food from the same plates, and sleeping close to each other in the same few huts at the centre of the compound rather than living scattered over an area of approximately 4 ha as in normal times (Figure 5). This, together with giving care to sick family members, was obviously not conducive for infection control. For a long time, the eldest son of the deceased head of the family refused to comply with the response team's advice to minimise contact within the family, and to disperse in the various family houses instead of gathering around the central ones. The notion of infection and transmission was rejected, instead the son was convinced that the family had been poisoned as immigrants by its autochthonous neighbours, and advised the other family members accordingly. The concept of poisoning is widespread in sub-Saharan Africa, and gained plausibility at this occasion by the fact that autochthonous neighbours who lived among the index family remained unaffected, and by the somewhat hostile reactions the index family experienced during the outbreak because of its Kenyan roots. Its siege mentality was probably further deepened by the sudden loss of the elders, which was, particularly in the traditional African context, a significant challenge to the family's social fabric.

Members of the response team from European, American, or African countries other than Uganda acted as "trusted strangers" and go-betweens for the index family and autochthonous neighbours. They were thus able to prevent escalation, for instance when they discouraged local surveillance offers from carrying out their duties under the protection of armed guards. It is interesting to consider the circumstances how the index family's attitude eventually became more cooperative. Despite daily visits by the outbreak response team, and considerable efforts to persuade the index family to follow the team's advice, the situation only improved after the death of the eldest son, when the family members eventually accepted to give up their daily congregations and to stay in their individual houses instead (Figure 5). The key intervention to change the family's attitude, however, was not undertaken by an epidemiologist or anthropologist, but by the driver of the surveillance vehicle: He held up the front page of a national newspaper ("New Vision", 6 Dec 2000), where the EHF death of a popular Ugandan doctor in Gulu made the headline "Ebola kills Dr. Lukwiya", and told the family: "Look: you are not the only ones who are affected!" It is difficult to plan for having the right intuition in the right moment - but flat hierarchies may help good ideas to emerge.

The general population of Masindi district, even at a considerable distance from the index family, was frightened. Panic reactions occurred, when a stampede was triggered by the rumour that an EHF patient wandered the hospital grounds: "100 patients flee Masindi Hospital over Ebola fear" was the newspaper headline of the day ("New Vision", 9 Dec 2000). Fear made it very difficult, at times impossible, to recruit community volunteers for activities like digging graves; international experts thus had to take on this role. Fear lead to acts of sabotage, when villagers filled in graves over night which the military had dug in advance. Fear resulted in discrimination, when HCWs found themselves banned from shops and market places. Fear gave rise to aggression, when a violent demonstration protested against the presence of EHF patients in Masindi hospital, accusing local politicians to accept them for money, or when HCWs found their house burned down after returning from work. Fear revived rivalries between Masindi and Kiryandongo inhabitants, when the former resented that patients from the index family were taken to Masindi hospital although Kiryandongo was closer by. Finally, fear alienated neighbours, who had lived peacefully with each other for decades, and locals demanded: "Those Kenyans who brought Ebola here - send them home!"

It is very important to be mentally prepared for such adverse community reactions, and to avoid anything which inflames the situation further. For instance, by the time health educators reach the villages, the community often knows already that EHF is a dangerous disease, so there is usually little point in stressing how dreadful and deadly it is. It is not helpful either to falsely state that "there is no treatment for Ebola", neglecting the availability of supportive treatment. Such statements discourage patients to accept isolation in the hospital, and can frequently be found in the media or in health education material. Instead, the community needs to be informed how its members can protect themselves, how EHF can be recognised in its early stage - particularly, that haemorrhage is not necessarily present -, and what to do when somebody falls ill with symptoms compatible with EHF. The community needs confidence instilled, not fear.

### Challenges in the health system

Recruiting public HCWs for the follow-up of contacts was difficult for fear of contamination; working in mobilisation teams in communities where transmission had not yet occurred was more popular. Even more difficult was the recruitment of clinical staff for work on the isolation unit for fear of occupational transmission of Ebola virus. The few HCWs who volunteered were quickly overworked. Staff meetings were called in, appealing to the solidarity of HCWs not to let down their volunteering colleagues. An agreement was reached resulting in more hospital staff working on the Ebola ward, but only for a few days each. While this strategy improved the situation, the resulting high turnover had two downsides: it put a considerable burden on expatriate staff for training a high number of hospital staff, and it prevented hospital staff from accumulating experience that would have had a positive effect on confidence and work safety.

Fear remained a major factor among HCWs. While more staff now agreed to work on the Ebola ward, not all of them trusted the protective gear and dared to get close enough to the patients for providing nursing care and supportive treatment. Oral rehydration is seen as an important component of supportive treatment [18]. However, when weak or confused patients lie in basic hospital beds without back support, they depend on others for helping them drink. Insufficient assistance for oral rehydration may have contributed to the initially high CFP, which with 76% was higher than what was observed in Gulu (51%, computed from own data and [3]) and other SEBOV outbreaks (Sudan 1976, 53% [1]; Sudan 1979, 65% [2]; Sudan 2004, 41% [6]). The much lower CFP after reinforcement arrived (20%) may be explained by improved quality of care, particularly improved rehydration, due to better staffing, reduced fear in and enhanced self-confidence in HCWs. This explanation is in line with observations of HCWs' practice on the ward. Alternative explanations for the difference in CFP include a lower viral load in occupational cases and virus attenuation.

HCWs' fears were sustained by the continuing occurrence of occupational transmission after the introduction of barrier-nursing. For most cases, breaches of barrier nursing by absent-minded or careless HCWs could be identified retrospectively. A recipe against absent-mindedness may be the 'buddy' system, which was introduced in later outbreaks, whereby each HCW touching a patient and becoming contaminated is accompanied by a second HCW who monitors the compliance with safety procedures and warns his buddy if a breach is imminent. Special attention should be given to the training and supervision of nursing aides, cleaners, drivers etc. When HCWs are careless and refuse to heed advice, they have to be prevented from working on the isolation ward for the safety of themselves and their colleagues.

Health care for EHF patients collapsed towards the end November, when the influx of patients increased drastically (9 patients within 3 days) and deaths from the virus increased including six HCWs and patients in the Ebola ward. These events revived fears and even panic among many staff members. Clinical staff stayed away from the Ebola ward, drivers absconded, and community volunteers could no longer be recruited, for digging graves (a no-risk activity) or as cleaners. When the crisis reached its maximum, there were four dead bodies on the Ebola ward for more than 48 h. Because body fluids tend to leak out of corpses in abundance, dead bodies are a significant source of contamination and must be disinfected, put into a body bag and buried swiftly. Furthermore, the prolonged presence of a dead body on the isolation ward is frightening and appalling and undermines the willingness of hospital staff to work on the Ebola ward and of probable cases, namely among HCWs, to accept hospitalisation. The nosocomial transmission of SEBOV from mother to child likely happened in this phase. Only the prevailing confusion can explain why the infant was not separated from its mother suffering from EHF.

At this point, the closure of the Ebola ward in Masindi, and the transfer of all patients to Gulu was considered by the District Ebola response team as last option. Fortunately, this measure was averted by the arrival of a high-level delegation from the MoH, and of a team from Gulu district consisting of senior international experts and experienced local staff. The direct interaction between high ranking MoH officials and the Masindi hospital staff boosted morale, while the increase in experts allowed reviving certain activities (e.g. public mobilisation). Gulu local staff turned the tide by fearlessly clearing the Ebola ward of dead bodies, thus acting as role models. With the approval of MoH the District Government paid out special allowances to attract and retain HCWs and volunteers involved in the Ebola response. Local hospital staff volunteered again to work on the Ebola ward and agreed to work there long enough to gather sufficient experience, and community members approached the response team asking whether they could join in the effort.

In retrospect it became apparent that the follow-up of contacts was not as comprehensive as it should have been. Patients who had been discharged from the 'probable' section of the Ebola ward as non-cases as well as staff working on the Ebola ward were not followed up systematically. In future outbreaks, the possibility of nosocomial and occupational transmission on the Ebola ward should be taken into account when establishing lists of contacts for follow-up.

For the VHF field laboratory to be fully supportive to the control efforts there must be an appropriate strategy for sampling, testing and communicating results. Isolation must not be delayed until the diagnosis has been laboratory confirmed for any length of time, as this would put contacts of probable cases at risk of transmission. Instead, probable cases must be isolated on clinical and epidemiological grounds alone, possibly in a holding area outside the isolation ward if that is more acceptable to patients. Given that the isolation ward is a very frightening place for most, probable cases who know that laboratory results will be available in a few days, notably HCWs who are probable cases themselves, may refuse isolation without laboratory confirmation. HCWs who decide on isolation may be less inclined to insist if the probable case is one of their colleagues. This occurred repeatedly during the Masindi outbreak, and at least one case of occupational Ebola infection occurred at Kiryandongo Hospital due to the delayed transfer of a probable case to the Ebola ward.

Antigen capture ELISA and PCR were the main tests to confirm current infection. At the time, the latter was believed to become positive one or two days earlier than the ELISA, which would be an obvious advantage to reduce in-hospital transmission between patients. On the other hand, positive PCR results repeatedly turned out to be false. This created considerable and unnecessary anxiety in the patients and put them at avoidable risk for in-hospital transmission. Specificity of PCR needed further improvement if the method was to become an essential tool for a BSL 4 field laboratory. Five years later, PCR was the dominant test during the Marburg HF outbreak in Uige/Angola [20].

The communication of laboratory results was problematic in two ways. Firstly, it was initially unclear who was responsible and entitled to receive laboratory results, so that conflicting information circulated, which resulted in an unnecessary burden on laboratory staff to respond to repeat queries. In the future, a medically qualified individual should be identified from the beginning to act as single contact person for the communication between laboratory and hospital. Secondly, oral communication by telephone in a setting where many family names are similar or identical and where many team members do not share the same mother tongue lead to several misunderstandings. This problem can be solved by using written communication by email, transmitted via telephone landlines, mobile phone networks, satellite connection or high frequency radio

### Limitations

Clinical records were not available for analysis. It was therefore almost impossible to investigate whether variations in the quality of supportive care may explain the striking differences in CFP between the two phases of the outbreak response. Two explanations have been offered for the records' absence. Firstly, it was reported that they were sent to Gulu to be analysed jointly with the clinical records from there. However, colleagues in Gulu denied having ever received them. In any case, clinical records from Masindi Ebola ward were property of Masindi hospitals, and should not have been removed; instead, copies should have been sent to Gulu. Secondly, it has been suggested that the possibly contaminated records may have been destroyed at the end of the epidemic to avoid that they could become the origin of renewed transmission, or that they are incriminated should such transmission occur from other origins. The risk that dry paper acts as fomite for the transmission of filovirus appears to be small given their susceptibility to drought and sunlight [21], but experiments could provide valuable data on viral survival rates on paper. Ultimately, guidelines on handling clinical records from Ebola wards should be agreed by the major players and approved by WHO to avoid unnecessary loss of data urgently needed to assess the effectiveness of treatment regimes for filoviral infections.

## Conclusions

In many ways, the response to the EHF outbreak in Masindi should have been straightforward: the number of cases was limited, all transmissions occurred in a controlled environment, a functioning hospital where an isolation ward could be set up was available and a FHF field laboratory within reach, and transport, communication and security were not particularly challenging compared with many other settings where FHF outbreaks have occurred. And yet, because of fear and resulting adverse reactions from local communities and HCWs, the response proved most challenging. This underlines once more the fundamental importance of establishing a relationship of trust and confidence with the families concerned, the community at large and local HCWs - such relationship is actually a necessary condition for a successful response [14]. Information management, i.e. providing authoritative information through a single and easily available source, is crucial in the prevention of perilous rumours.

Since the first occurrence of FHF outbreaks in their natural environment in 1976, a vast amount of knowledge has been accumulated on how such outbreaks should be investigated and responded to. However, a painful gap persists until today: how best to treat FHF disease. The EHF outbreak in Masindi was another missed opportunity in this respect: all clinical records were lost or destroyed. Thus, we can only suggest, based on observations, that intense supportive treatment may improve survival. The dearth of clinical data has reached a stage where the failure to contribute to our understanding of best clinical practice should be considered as unethical. We urgently need a consensus on which clinical data ought to be documented, and how [22].

## Competing interests

The authors declare that they have no competing interests.

## Authors' contributions

MB contributed to outbreak investigation and response, conducted the data analysis, and drafted the manuscript. IM, JL, HL, GB, JT, PP, and NN contributed to outbreak investigation and response and reviewed the manuscript critically. MVK contributed to interpreting results and drafting the manuscript. PR contributed to interpreting results and reviewed the manuscript critically. PVDS contributed to developing the data analysis plan and to interpreting results, and reviewed the manuscript critically. All authors read and approved the final manuscript.

## Authors' information

MB, a medical epidemiologist with a particular interest in FHF, contributed to the Masindi EHF response as consultant for the Ministry of Health, and on two occasions (Watsa/DRC and Uige/Angola) to Marburg HF response activities as volunteer for Médecins Sans Frontières. IM and JT were, during the EHF outbreak, Medical Superintentends of Masindi and Kiryandongo hospitals, respectively. MVK is infectious disease epidemiologist with a special interest outbreak investigations, and whose research interests include avian influenza, pandemic influenza and FHF. JL is virologist and involved in FHF diagnostics at the Uganda Virus Research Institute. HL and GB were, during the EHF outbreak, Director and Deputy Director of Health Services in Masindi district. PP contributed to the Masindi EHF response as clinician for Médecins Sans Frontières, ND as epidemiologist for WHO-AFRO. PR is epidemiologist with Médecins Sans Frontières and has participated in the response to several FHF outbreaks. PVDS is senior epidemiologist whose research interests include FHF and Dengue fever.

## Pre-publication history

The pre-publication history for this paper can be accessed here:

http://www.biomedcentral.com/1471-2334/11/357/prepub
